# Differential expression of diacylglycerol kinase ζ is involved in inferior parietal lobule-related dysfunction in schizophrenia with cognitive impairments

**DOI:** 10.1186/s12888-023-04955-x

**Published:** 2023-07-21

**Authors:** Xiao-Fan Liu, Shu-Wan Zhao, Jin-Jin Cui, Yue-Wen Gu, Jing-Wen Fan, Yu-Fei Fu, Ya-Hong Zhang, Hong Yin, Kun Chen, Long-Biao Cui

**Affiliations:** 1 Department of Radiology, Xi’an People’s Hospital, Xi’an, China; 2 Schizophrenia Imaging Lab, Fourth Military Medical University, Xi’an, China; 3 Department of Radiology, The Second Medical Center, Chinese PLA General Hospital, Beijing, China; 4 Department of Psychiatry, Xijing Hospital, Fourth Military Medical University, Xi’an, China; 5 Department of Human Anatomy and K. K. Leung Brain Research Centre, Fourth Military Medical University, Xi’an, China; 6 Shaanxi Provincial Key Laboratory of Clinic Genetics, Fourth Military Medical University, Xi’an, China; 7 Department of Radiology, The First Affiliated Hospital of Xi’an Jiaotong University, Xi’an, China

**Keywords:** Inferior parietal lobule, Schizophrenia, Cognitive function, Transcriptome

## Abstract

**Background:**

Cognitive impairment is the main factor in the poor prognosis of schizophrenia, but its mechanism remains unclear. The inferior parietal lobule (IPL) is related to various clinical symptoms and cognitive impairment in schizophrenia. We aimed to explore the relationship between IPL-related functions and cognitive impairment in schizophrenia.

**Methods:**

136 schizophrenia patients and 146 demographically matched healthy controls were enrolled for a cross-sectional study. High-spatial-resolution structural and resting-state functional images were acquired to demonstrate the alternations of brain structure and function. At the same time, the digit span and digit symbol coding tasks of the Chinese Wechsler Adult Intelligence Test Revised (WAIS-RC) were utilized in assessing the subjects’ cognitive function. Patients were divided into cognitive impairment and normal cognitive groups according to their cognitive score and then compared whether there were differences between the three groups in fractional amplitude of low-frequency fluctuation (fALFF). In addition, we did a correlation analysis between cognitive function and the fALFF for the left IPL of patients and healthy controls. Based on the Allen Human Brain Atlas, we obtained genes expressed in the left IPL, which were then intersected with the transcriptome-wide association study results and differentially expressed genes in schizophrenia.

**Results:**

Grouping of patients by the backward digit span task and the digit symbol coding task showed differences in fALFF values between healthy controls and cognitive impairment patients (*P* < 0.05). We found a negative correlation between the backward digit span task score and fALFF of the left IPL in healthy controls (*r* = − 0.388, *P* = 0.003), which was not seen in patients (*r* = 0.203, *P* = 0.020). In addition, none of the other analyses were statistically significant (*P* > 0.017). In addition, we found that diacylglycerol kinase ζ (*DGKζ*) is differentially expressed in the left IPL and associated with schizophrenia.

**Conclusion:**

Our study demonstrates that the left IPL plays a vital role in cognitive impairment in schizophrenia. *DGKζ *may act as an essential regulator in the left IPL of schizophrenia patients with cognitive impairment.

**Supplementary Information:**

The online version contains supplementary material available at 10.1186/s12888-023-04955-x.

## Introduction

Schizophrenia is a chronic mental disorder with an undefined aetiology and is highly recurrent and extremely disabling, affecting more than 20 million people worldwide [1]. The Chinese Mental Health Survey showed schizophrenia has a weighted lifetime prevalence of 0.6% [2]. The clinical diagnosis and treatment for this disorder have progressed slowly, thus creating a massive healthcare burden. As a symptom dimension of the disease, cognitive impairment is one of the critical clinical problems and difficulties of schizophrenia and a significant factor in the poor prognosis of schizophrenia [3]. There is no effective treatment method for cognitive impairment, and the root is that the mechanism has not been fully elucidated [4–6].

Magnetic resonance imaging (MRI) is vital in revealing brain structure and function information in schizophrenia patients. In fMRI studies of schizophrenia, many researchers have devoted themselves to finding the functional localization of brain regions corresponding to specific symptoms, including cognitive symptoms [7]. The adoption of MRI contributes to systematic study on the brain of schizophrenia patients to explore the mechanisms of cognitive impairment in schizophrenia.

Both amplitude of low-frequency fluctuation (ALFF) and fractional amplitude of low-frequency fluctuation (fALFF) are indicators of the intensity of spontaneous activity in the brain. ALFF has some drawbacks due to noise [8]. The fALFF effectively avoids the drawbacks of ALFF by calculation. It has been shown that fALFF is more sensitive to cognitive domain differences [9]. Therefore, we detected the fALFF function in patients. The inferior parietal lobule (IPL) is a crucial component of the frontoparietal network and is involved in cognitive deficits and various clinical symptoms in schizophrenia. IPL involves sensorimotor integration [10], semantic processing [11], mathematical cognition [12], body image [13], the concept of self [13], and execution [14]. Many studies have reported the structural alterations of IPL in schizophrenia, such as reduced grey matter volume [15]. There is a relationship between brain morphology and cognitive function [16]. A meta-analysis has shown that reduced gray matter volume in the IPL is associated with cognitive function [17]. Similarly, it has also found the abnormal function of IPL in patients with schizophrenia [18–20]. However, it is unclear whether changes in IPL function are correlated with cognitive impairment in patients with schizophrenia. In addition, the molecular genetic mechanisms behind the large-scale mechanisms of cognitive impairment in schizophrenia remain to be clarified. Common brain phenotypic abnormalities in schizophrenia include functional abnormalities between brain regions [21]. Alterations in brain function are associated with schizophrenia risk genes on the one hand [22] and with cognitive function on the other hand [23].

**Fig. 1:**
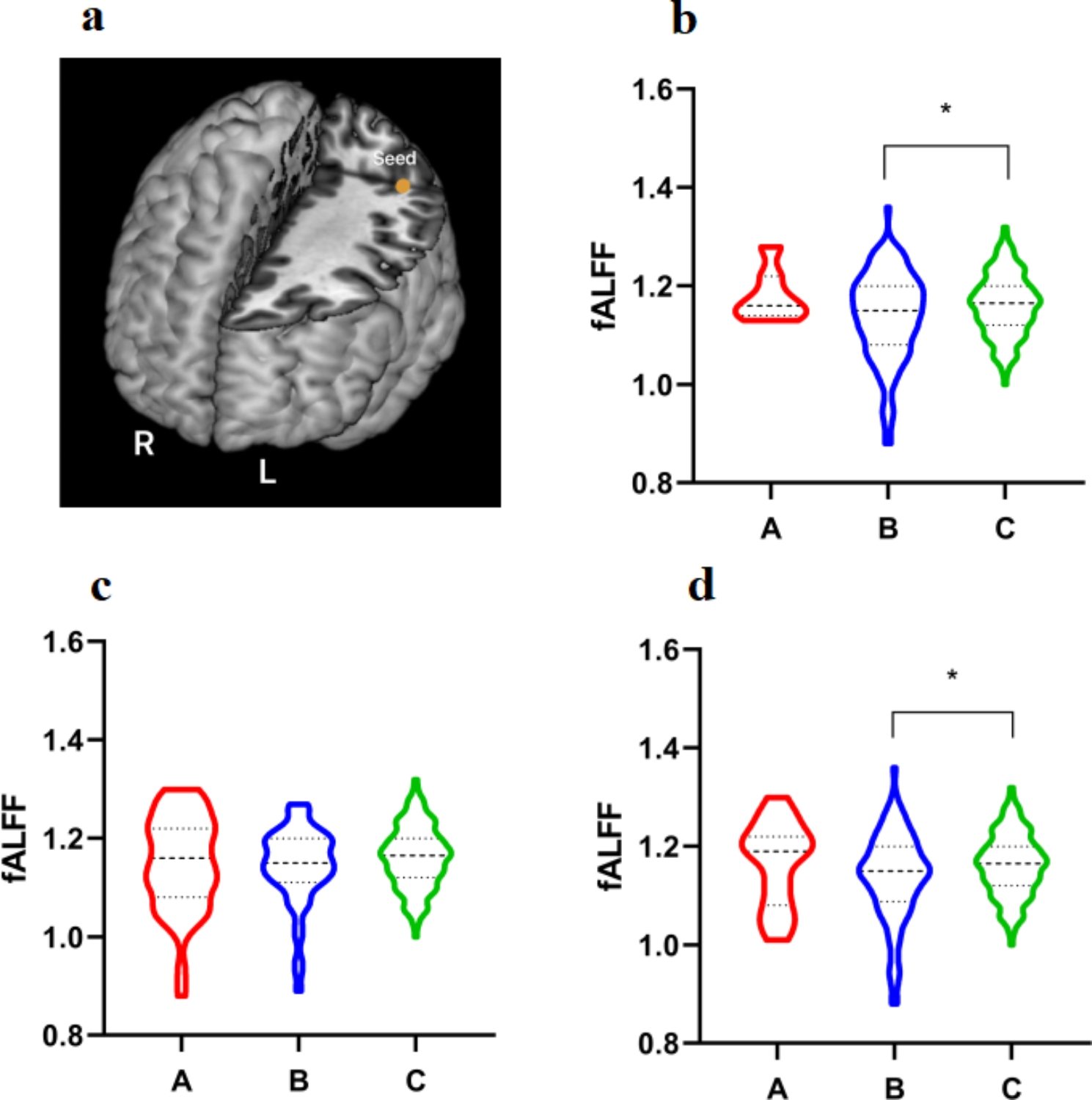
**a**, The impairment of left IPL function may play an essential role in the cognitive impairment of schizophrenia. **b**, Using the digit symbol coding task scores for grouping, the difference in fALFF values for the left IPL was statistically significant between healthy controls and patients with cognitive impairment (*P* = 0.014). **c**, Using the forward digit span scores for grouping, the group difference has no statistical significance (*P* = 0.259). d, Using the backward digit span scores for grouping, the difference in fALFF values for the left IPL was statistically significant between healthy controls and patients with cognitive impairment (*P* = 0.014). (**A**: normal cognitive group; **B**: cognitive impairment group; **C**: healthy control)

The current study examined the left IPL local function and its relevance to cognitive function. To explore the functional connectivity abnormalities, we obtained resting-state fMRI data from patients with schizophrenia and healthy controls. Comparing patients with healthy controls, we intended to investigate the relationship between IPL and cognitive impairment in patients with schizophrenia. (Fig. 1). In addition, there is a need to explore the molecular mechanisms behind cognitive impairment in schizophrenia.

## Materials and methods

### Subject

We recruited 136 schizophrenia patients from the Department of Psychiatry at Xijing Hospital and 146 healthy controls through advertising for a cross-sectional study. One hundred and nine patients were on medication converted to olanzapine equivalents with a mean (standard deviation) of 9.50 mg/d (6.94 mg/d), including that six were on both first- and second-generation antipsychotics, and the others were on second-generation antipsychotics. All subjects signed informed consent. The Xijing Hospital Institutional Ethics Committee approved the study, which followed the principles in the Declaration of Helsinki. All subjects were Han Chinese and right-handed. Exclusion criteria included (1) organic lesions of the central nervous system; (2) history of neurostimulation, congenital brain development or head trauma, epilepsy, etc.; (3) other types of mental disorders (such as obsessive-compulsive disorder, depression, anxiety disorder, etc.); (4) severe unstable somatic diseases (such as coronary heart disease, systemic lupus erythematosus, thyroid disease, hypertension, etc.); (5) substance abuse or substance dependence; (6) pregnancy or preparation for pregnancy, lactation; (7) contraindications for MRI examination (e.g., pacemaker and other metal implants, etc.). A full description of the sample is available in the study by Li et al. [9].

The diagnosis of schizophrenia patients follows the schizophrenia diagnostic criteria in the Diagnostic and Statistical Manual of Mental Disorders, Fifth Edition (DSM-5) [24]. Two experienced clinical psychiatrists reached an agreement after an independent diagnosis. The severity of patients’ symptoms was evaluated by using the Positive and Negative Syndrome Scale (PANSS) [25]. Clinical assessment and MRI scans were completed on the same day.

### Cognitive assessment

We assessed the cognition of subjects with the digit span (forward, backward) and digit symbol coding tasks in the Wechsler Adult Intelligence Scale-Revised in China (WAIS-RC). The forward digit span task consists of up to 12 digits, while the backward digit span task consists of up to 10 digits, with each part arranged from easy to difficult digits. If a string is repeated correctly, subjects are asked to proceed to the next string. If there is an error, the second test uses a new string of the same length. The test stops for the part that all fails, and the string’s length is recorded. In the digit symbol coding task, the number 0 to 9 corresponds to a specified symbol, and the subject is required to quickly fill in the corresponding symbol in the space under each number from left to right. Subjects start with the practice program. The time limit of formal test is 90 s. Ultimately, we obtained data on digit symbol coding and digit span task from 132 patients and 56 healthy controls.

### Image acquisition

Resting-state fMRI and structural T1-weighted imaging scans were collected for each subject on a General Electric (GE) Discovery MR750 3.0 T MR. During acquiring the images, participants were asked to lie flat, remain still, close their eyes, and not fall asleep. The custom head ring pads and ear plugs were used to reduce head movement and weaken the scanner’s noise.

### Data preprocessing

As in the previous study, we processed the data through the Data Processing Assistant for Resting-State fMRI Advanced Edition (DPARSFA) V4.4 [9]. The steps were: (1) converting the DICOM image format to NIfTI; (2) removing the first ten time points for each subject; (3) slice timing correction; (4) realignment; (5) the regression of covariates, including global mean signal, six head motion parameters, white matter signal, and cerebrospinal fluid signals; (6) coregistering the T1-weighted images to the functional images; (7) normalizing coregistered images to the Montreal Neurological Institute space; (8) smoothing.

### ALFF calculation

The ALFF of the BOLD signal was performed using DPARSFA V4.4 to identify regional brain function [26]. The data were filtered by bandpass (0.01–0.08 Hz) for ALFF. fALFF was the ratio of the power of each frequency in the low-frequency range to the power of the entire frequency range.

### Gene expression

A full description of RNA-seq data collection and analysis is available elsewhere [27] Total RNA was extracted using the Trizol reagent kit. Eukaryotic mRNA was subsequently enriched by Oligo(dT) beads, while prokaryotic mRNA was enriched by the removal of rRNA with the Ribo-ZeroTM magnetic kit. The enriched mRNA was then broken into short fragments using a broken buffer and reverse transcribed into cDNA using random primers. Second strand cDNA was synthesized using DNA polymerase I, RNase H, dNTP and buffer. cDNA fragments were then purified, end-repaired, poly(A) added, and ligated to Illumina sequencing adapters. Finally, PCR amplification and sequencing used Illumina Novaseq6000. We searched through a web Allen Human Brain Atlas (http://human.brain-map.org/) and acquired the genes expressed in IPL. Then, we compared the transcriptome-wide association study (TWAS) results of schizophrenia [28] and differentially expressed genes in schizophrenia [27] with the genes expressed in IPL.

### Statistical analysis

We used WAIS-RC scores to describe cognitive grouping, i.e., a cutoff of scores of < − 1.0 SD to describe the group with cognitive impairment, and scores of ≥ − 0.5 SD to describe a group with normal cognitive function [29]. Then we compared the differences between the three groups in fALFF using the one-way ANOVA test. In addition, we made the Pearson correlation analysis between ALFF of the left IPL of patients and healthy controls and digit symbol coding task score, forward digit span task score, and backward digit span task score. To verify whether positive symptoms could affect cognitive scores, we did a Pearson correlation analysis between positive symptom scores and digit symbol coding task score, forward digit span task score, and backward digit span task score. In addition, Pearson correlation analysis between medication dose and cognitive scores was performed in patients with schizophrenia. *P* < 0.05 was considered statistically significant.

## Results

### Demographical and clinical characteristics

Demographic data are shown in Supplementary Table 1. There were no statistically significant differences between schizophrenia patients and healthy controls on characteristics other than educational attainment.

### Cognitive Impairments

Grouping of patients by the backward digit span task and the digit symbol coding task showed differences in fALFF values between healthy controls and cognitive impairment patients (*P* < 0.05). All other differences analyzed were not statistically significant (*P* > 0.05).

### Clinical correlates

**Fig. 2:**
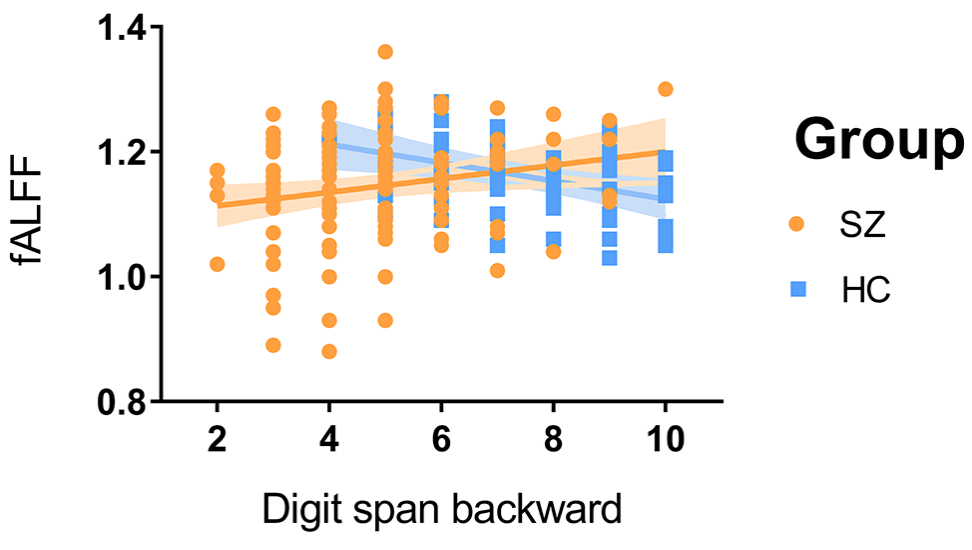
There was a negative correlation between digit span task score and fALFF in the left IPL in healthy controls (*r* = − 0.388, *P* = 0.003), but was not seen in patients (*r* = 0.203, *P* = 0.020)

The results of the subgroups of patients with schizophrenia are shown in Table 1. We made the correlation analysis between ALFF of the left IPL (x = − 38, y = − 58, z = 38, radius = 6 mm) [30] of patients and healthy controls and digit symbol coding task score, forward digit span task score, and backward digit span task score in WAIS scale. The results showed a negative correlation in healthy controls (*r* = -0.388, *P* = 0.003) but were not seen in patients (*r* = 0.203, *P* = 0.020; Fig. 2). In addition, none of the other correlation analyses was statistically significant (*P* > 0.05, Bonferroni correction, 0.05/3). The above findings demonstrate that the cognitive impairment in schizophrenia patients is obvious, and the function of the left IPL involved in working memory is impaired in patients with schizophrenia. In addition, the results of the correlation analysis between positive symptom scores and digit symbol coding and digit span scores were not statistically significant, indicating that positive symptoms do not affect digit symbol coding and digit span results. The results of the correlation analysis between medication dose and cognitive scores in schizophrenia patients were not statistically significant.

**Table 1:** Results of subgroups of patients with schizophrenia

	normal cognitive groups	cognitive impairment groups
digit symbol coding	7	114
digit span (forward)	43	57
digit span (backward)	19	98

### Gene expression

**Fig. 3:**
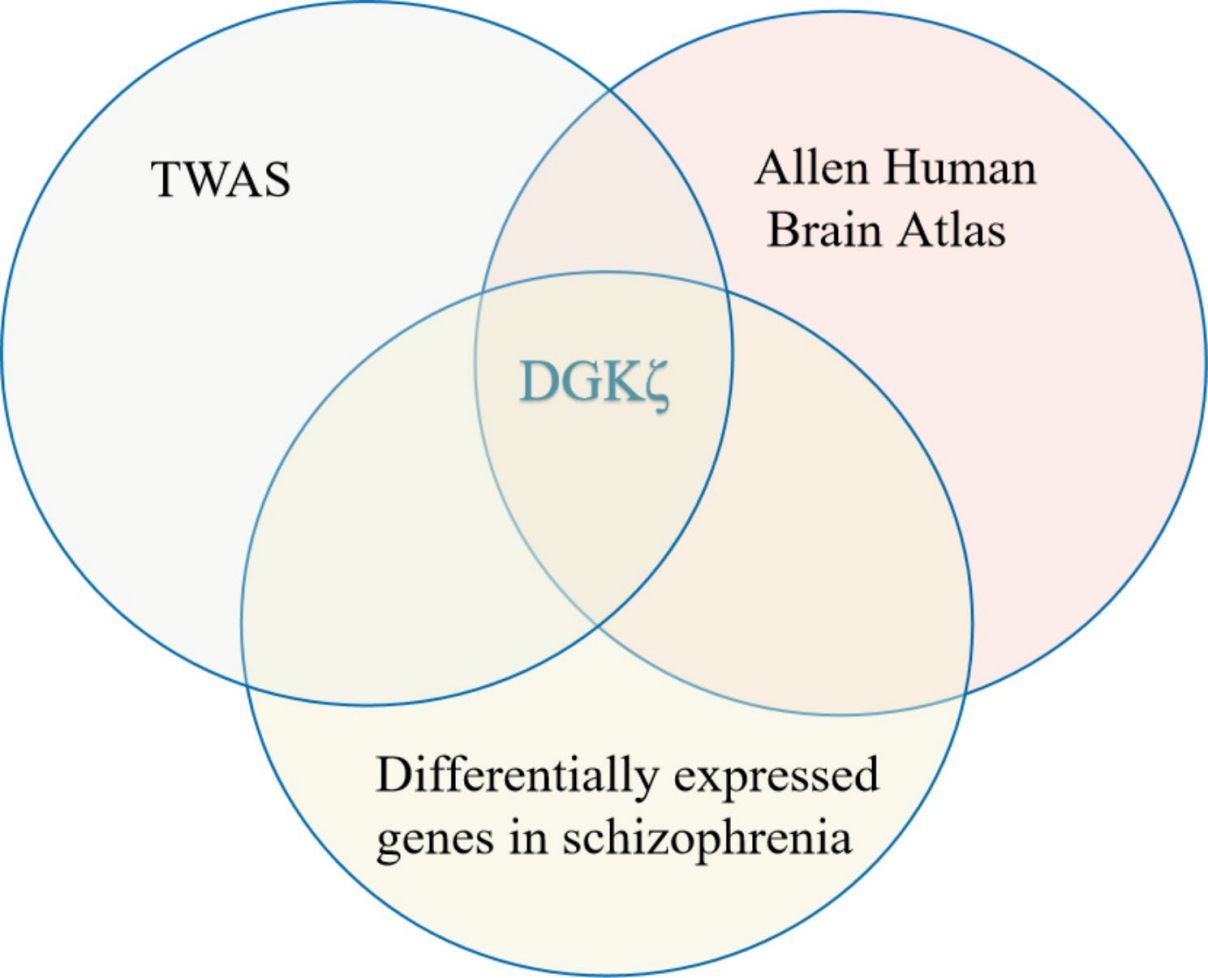
The TWAS results of schizophrenia [28], differentially expressed genes in schizophrenia, and the genes expressed in IPL were intersected. *DGKζ* was differentially expressed in the IPL and associated with schizophrenia

*DGKζ* was differentially expressed in the left IPL and associated with schizophrenia (Fig. 3).

## Discussion

**Fig. 4:**
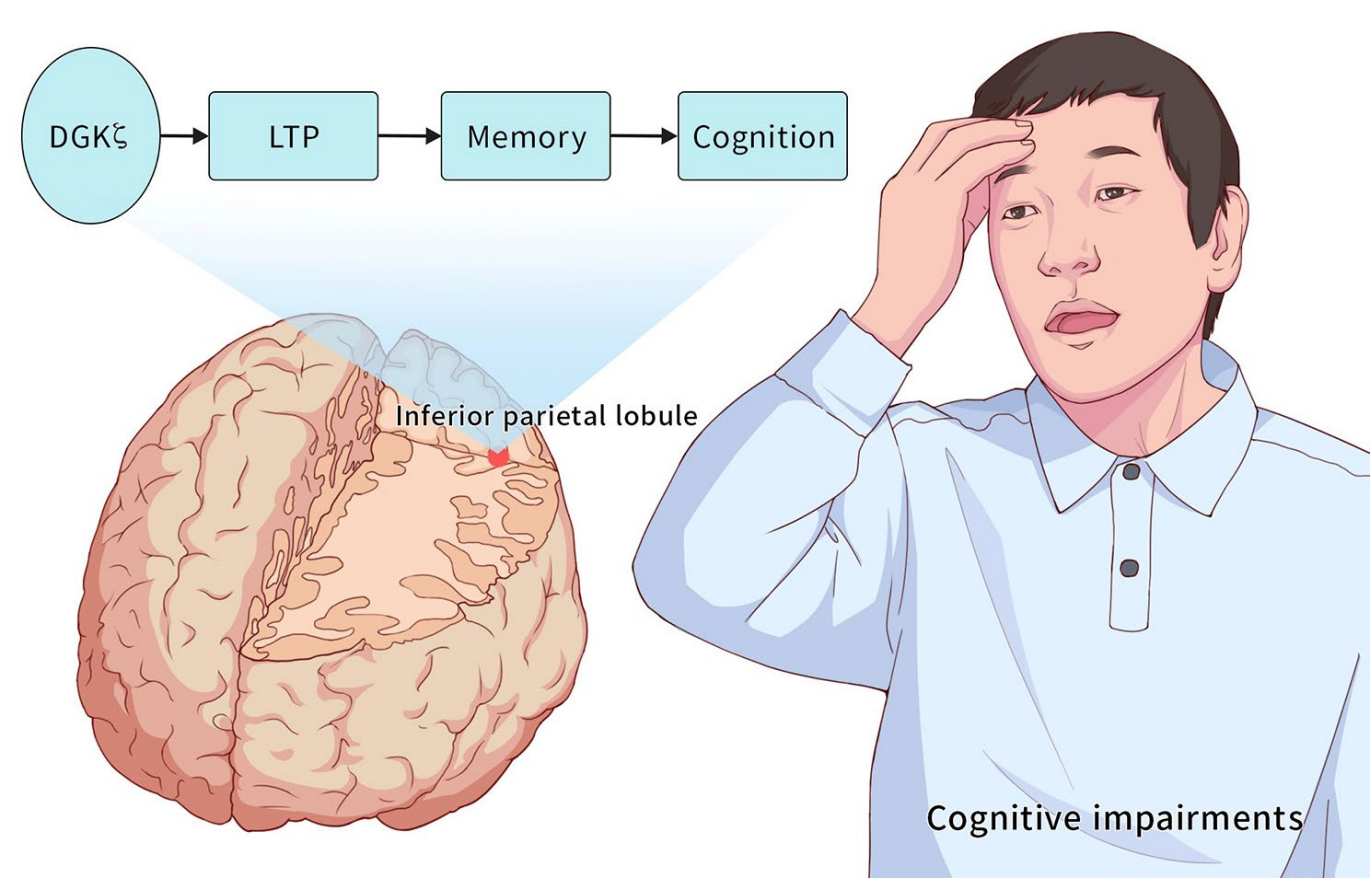
Disrupted IPL function may play a vital role in cognitive impairment in schizophrenia. *DGKζ* may act as an essential regulator in the left IPL of schizophrenia patients with cognitive impairment

Our study explores the relationship between IPL dysfunction and cognitive impairment in schizophrenia. The results show that IPL functional impairment may work on cognitive impairment in schizophrenia (Fig. 4). In addition, *DGKζ* is differentially expressed in the left IPL and associated with schizophrenia.

Regional abnormalities in the frontoparietal network, the hub node of the brain connectome, have been associated with cognitive impairment in schizophrenia [29, 31–33], especially the connection between the DLPFC-IPL [31]. Although the parietal cortex, especially the IPL, is generally considered an essential component of network disorders in schizophrenia, compared with the hippocampus, prefrontal cortex, or cingulate gyrus, IPL has received less attention [13]. fMRI study has shown that patients with cognitive impairment display decreased frontoparietal network connectivity and that higher ALFF of IPL corresponds to worse cognitive function [29]. Multimodal studies have also indicated that most brain regions with dysfunctions and structural abnormalities associated with cognitive impairment typically contain IPL.

Our findings demonstrate the relationship between the dysfunction of left IPL and cognitive impairment, prove that IPL has a significant role in the cognitive impairment of schizophrenia, and reveals the biological phenotype of schizophrenia. In addition, the conclusions also provide evidence that the left IPL can be used as a new target for noninvasive neurostimulation for cognitive impairment in schizophrenia, which can help to optimize the current clinical treatment strategies.

In the present study, cognitive assessments were made using the digit span (forward, backward) and digit symbol coding tasks. In the digit symbol coding and backward digit span tasks, we found differences in fALFF in the left IPL between healthy controls and cognitive impairment patients. This suggests that the left IPL of cognitive impairment patients has impaired functions involved in working memory and processing speed. Previous studies have shown that patients with schizophrenia are more likely to have impaired working memory [34] and processing speed [35]. However, subgroup studies of patients have yet to be conducted. The digit symbol coding task is sensitive to schizophrenia [36] and reflects processing speed. The digit symbol coding task score is a characteristic indicator of cognitive deficits in schizophrenia. Koshiyama et al. investigated the relationship between brain structure and cognitive function in patients with schizophrenia. They showed a positive correlation between the volume of the right nucleus accumbens and the digit symbol coding task scores in the group of patients [37]. The digit span test reflects working memory. Moreover, memory and schizophrenia cognition are closely related. There is a significant association between cortical thickness in frontoparietal regions and digit span score in children [38]. Although previous studies have focused on the association between frontal lobes and working memory in schizophrenia, the relevance of parietal lobes to working memory remains largely unknown.

A growing body of evidence supports the multifactorial nature of schizophrenia susceptibility, which includes smoking, obesity, inflammation, and genetic factors. Smoking may increase the risk of developing schizophrenia [39]. Moreover, smoking rates are higher in schizophrenia. Molecular genetic studies have revealed a common genetic root of schizophrenia and smoking [40]. Inflammatory processes are known to play a role in the etiology of schizophrenia. One study found high plasma resistin levels in both first-episode schizophrenia patients and chronic schizophrenia patients, suggesting a role for resistin in the inflammatory process in both acute and chronic phases of psychosis [41]. However, previous findings do not support the idea that inflammation plays a major role in cognitive impairment in schizophrenia [42]. Adolescents with psychiatric disorders have unhealthy lifestyles, which may contribute to increased obesity rates [43]. However, some studies suggest that obesity in schizophrenia is primarily caused by antipsychotic medications [44]. It has also been suggested that schizophrenia itself can increase the body mass index (BMI), leading to obesity [45]. However, it has not found differences in BMI between patients with schizophrenia and healthy controls [46]. In addition, oxidative mechanisms may also play a role in the pathogenesis of schizophrenia, with one study finding a correlation between elevated total antioxidant levels and suicidal behavior [47].

Studies have shown that *DGKζ* is critical for immune response [48]. Studies on the potential role of inflammation in the etiology of schizophrenia have been widely reported. Studies have shown that the inflammatory process might destroy cognition’s neurobiological mechanism [49]. In addition, Müller et al. [50] concluded that anti-inflammatory add-on risperidone could improve the cognition of schizophrenia patients. Therefore, our study indicates that *DGKζ* may be engaged in the molecular mechanism of function impairment in IPL through immunomodulatory function and serve an essential role in the cognitive impairment of schizophrenia.

In addition, it has been shown that *DGKζ* is a vital regulator of long-term depression and long-term potentiation (LTP) in the hippocampus, and *DGKζ* may be a negative regulator of LTP under physiological conditions [51]. LTP is the physiological basis of memory. In schizophrenia, memory and cognitive function are closely related. LTP may be related to the neuropathological mechanism of cognitive impairment in schizophrenia [52]. Therefore, *DGKζ* may affect the cognitive function of schizophrenia by regulating LTP.

There are some limitations of our study. First, our study concentrated on a limited period during the illness without studying the different stages of schizophrenia. Several aspects of the current study deserve further discussion, and future work should include the normal cognitive group of patients with schizophrenia for multi-group comparisons. Second, the lack of relevant information made it impossible to explore the effects of extrinsically influenced factors on the current findings. Therefore, relevant data should be added for analysis in future studies. The molecular mechanism of IPL functional alterations leading to cognitive impairment in schizophrenia deserves further exploration. Furthermore, physical stimuli, such as electroconvulsive therapy and repetitive transcranial magnetic stimulation, can be performed to detect the effect of activating left IPL on cognitive impairment, thus demonstrating that the left IPL plays a vital role in cognitive impairment in schizophrenia.

In summary, the dysfunction of left IPL may play an essential role in the cognitive impairment of schizophrenia. The differential expression of *DGKζ* may be involved in IPL-related dysfunction in schizophrenia with cognitive impairments.

## Electronic supplementary material

Below is the link to the electronic supplementary material.

Supplementary Material 1

## Data Availability

The data underlying this article will be shared on reasonable request from the corresponding author.
