# Supplementary material for: Differential expression of diacylglycerol kinase ζ is involved in inferior parietal lobule-related dysfunction in schizophrenia with cognitive impairments
Source: BMC Psychiatry. 2023 Jul 21;23:526. doi: 10.1186/s12888-023-04955-x (PMC10362743; doi:10.1186/s12888-023-04955-x)
Supplement: Supplementary file 1 — Supplementary Material 1 [file 12888_2023_4955_MOESM1_ESM.docx]

Supplementary Table1 Demographical and clinical characteristics.

|  | **Schizophrenia patients**  **(n = 136)** | **Healthy controls**  **(n = 146)** | ***p*-values** |
| --- | --- | --- | --- |
| Age, y^a^ | 24.1 (7.4) | 24.2 (5.2) | 0.922 |
| Gender, M/F^b^ | 75/61 | 82/64 | 0.864 |
| Education, y^a^ | 12 (3) | 15 (3) | <0.001 |
| Status, FE/NFE | 101/35 | / |  |
| Medication, U/T | 27/109 | / |  |
| Illness duration, mon | 14.7 (22.7) | / |  |
| PANSS score |  |  |  |
| Positive | 21.7 (5.3) | / |  |
| Negative | 20.2 (7.3) | / |  |
| General | 43.7 (8.3) | / |  |
| Total | 85.6 (14.3) | / |  |

*Data are shown in mean (standard deviation).*

*FE, fifirst episode; NFE, non-first episode; U, untreated; T, treated.*

*^a^ Two-sample t-test.*

*^b^Pearson Chi-Square test.*
